# Thymic large cell neuroendocrine carcinoma – a rare and aggressive tumor: a case report

**DOI:** 10.1186/s13256-017-1331-2

**Published:** 2017-06-12

**Authors:** Efared Boubacar, Gabrielle Atsame-Ebang, Sani Rabiou, Ammor Fatimazahra, Asmae Mazti, Ibrahim S. Sidibé, Layla Tahiri, Nawal Hammas, Ouadnouni Yassine, Smahi Mohamed, Chbani Laila, El Fatemi Hinde

**Affiliations:** 1 Department of Pathology, Hassan II University Hospital, Fès, Morocco; 2 Department of Thoracic Surgery, Hassan II University Hospital, Fès, Morocco; 3 Faculty of Medicine and Pharmacology, Sidi Mohamed ben Abdellah University, Fès, Morocco

**Keywords:** Thymus, Neuroendocrine tumors, Large cell neuroendocrine carcinoma, Pathology

## Abstract

**Background:**

Neuroendocrine tumors are a large group of tumors with a wide spectrum of behavior, affecting mainly the digestive system and the lung. The thymus is very rarely affected.

**Case presentation:**

A 28-year-old Arab woman presented with chronic chest pain and dyspnea. A computed tomography scan showed a huge anterior mediastinal mass invading neighboring structures. A mediastinotomy was performed with biopsies of the mass. Pathological findings were consistent with a thymic large cell neuroendocrine carcinoma.

**Conclusions:**

The occurrence of a large cell neuroendocrine carcinoma in the thymus, especially in young people, is extremely rare. In this current report, we discuss the clinicopathological issues of this rare tumor according to recent literature data.

## Background

Neuroendocrine tumors (NETs) are a group of tumors affecting mainly the digestive system and the respiratory system but organs like the thymus are rarely affected [1–3]. They constitute a wide spectrum of neoplasms with a variety of prognoses relying on a constellation of some histological features used to grade them [1, 2, 4]. The last World Health Organization (WHO) classification of tumors of the lung, pleura, thymus, and heart, categorized NETs into two major prognostic groups. One major prognostic group is low and intermediate grade tumors: typical and atypical carcinoid respectively. The other major group is high grade tumors: small cell neuroendocrine carcinoma (SCNEC) and large cell neuroendocrine carcinoma (LCNEC) [1].

Thymic NETs are very rare, representing approximately 2 to 5% of all thymic neoplasms [1, 2]. The majority of thymic NETs are carcinoid tumors, high grade tumors are not encountered as often. LCNECs account for 14 to 26% of all NETs of the thymus with a median age at presentation around 50 years [1]. The LCNEC is a tumor with a very poor prognosis as most patients present at an advanced stage of the disease with local and distant spreading [1, 5, 6]. However, little is known about NETs of the thymus owing to their rarity. This fact leads to their assimilation with their pulmonary counterparts despite some differences regarding their epidemiological, clinical, and histopathological features compared to lung NETs [1–6].

Here we report a case of a 28-year-old woman who did not smoke tobacco presenting with a locally advanced thymic LCNEC, with discussion about clinical and histopathological features according to the literature review.

## Case presentation

A 28-year-old Arab woman presented to our hospital with a 3-year history of chronic chest pain. She described recent worsening of symptoms with the onset of dyspnea and a pain radiating to her neck and to her left arm. She had no history of tobacco smoking; she reported a history of salpingectomy for ectopic pregnancy 2 years ago. She had no particular familial history. She presented with weight loss and a loss of appetite. On physical examination a decrease in breath sounds and vocal vibrations was noted in the left side of her chest wall. The rest of her physical examination was normal.

**Fig. 1:**
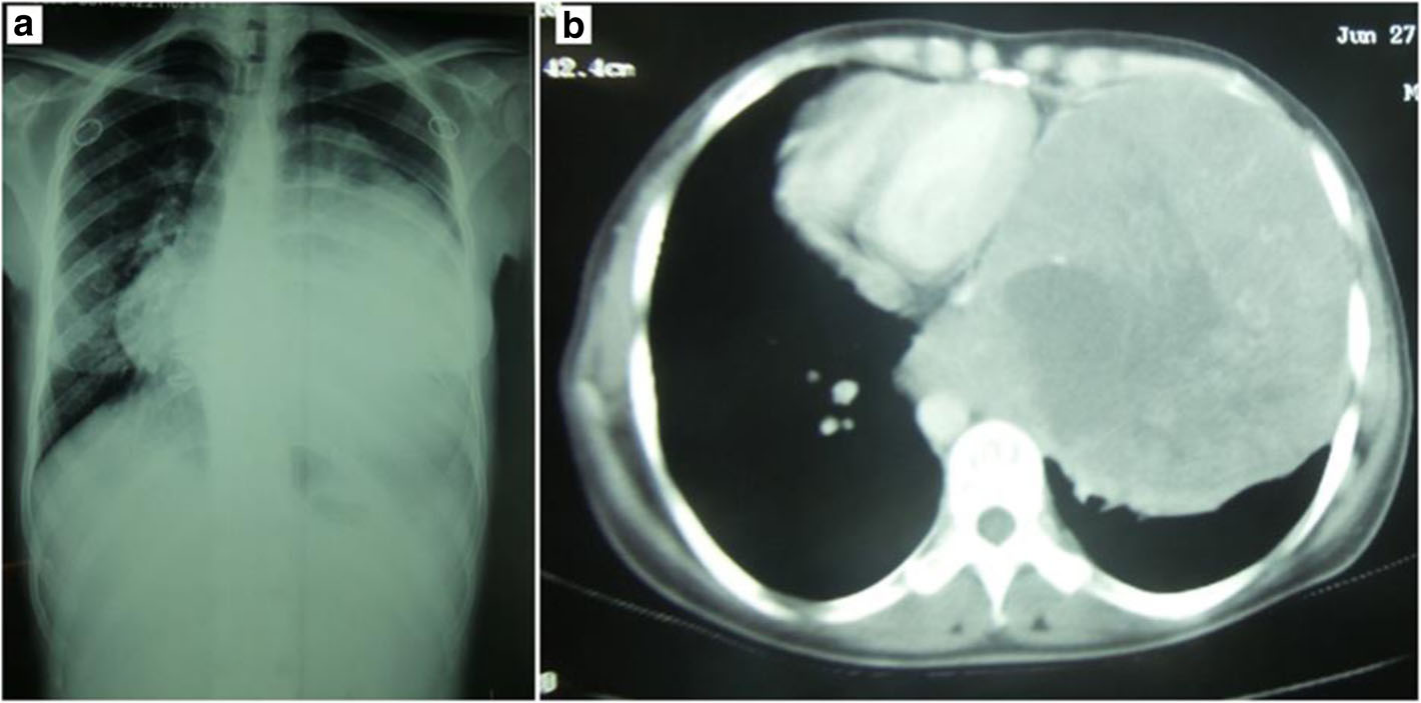
A chest X-ray shows a homogenous left-sided opacity occupying almost the entire left hemithorax, with non-visible left diaphragmatic dome (**a**). A chest computed tomography scan reveals an enormous mediastinal mass with a central area of necrosis and compression of mediastinal structures towards the right side (**b**)

Her blood tests were within normal limits. A chest X-ray (Fig. 1a) showed a homogenous left-sided opacity occupying almost her entire left hemithorax.

A chest computed tomography (CT) scan (Fig. 1b) revealed an enormous mediastinal mass with a central area of necrosis and compression of mediastinal structures towards the right side.

**Fig. 2:**
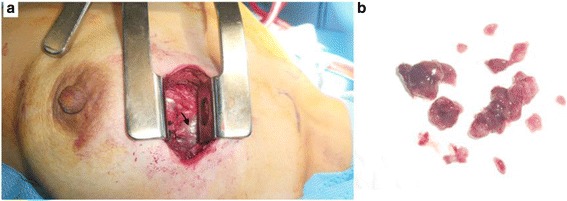
A perioperative view of the tumor showing an anterior mediastinal *pink-colored* mass with a smooth contour (**a**, *black arrow*). Biopsy sample of the tumor shows *pink* fragments with areas of hemorrhagic changes (**b**)

Given the radiological aspect of the tumor, an anterior left mediastinotomy was performed. It showed a tumoral mass in the anterior mediastinum that adhered to neighboring mediastinal structures and seemed to invade her left lung. The tumor was pink-colored with a smooth surface (Fig. 2a). Because of these adherences to mediastinal structures a surgical option was not considered and a biopsy of the tumor was performed (Fig. 2b).

**Fig. 3:**
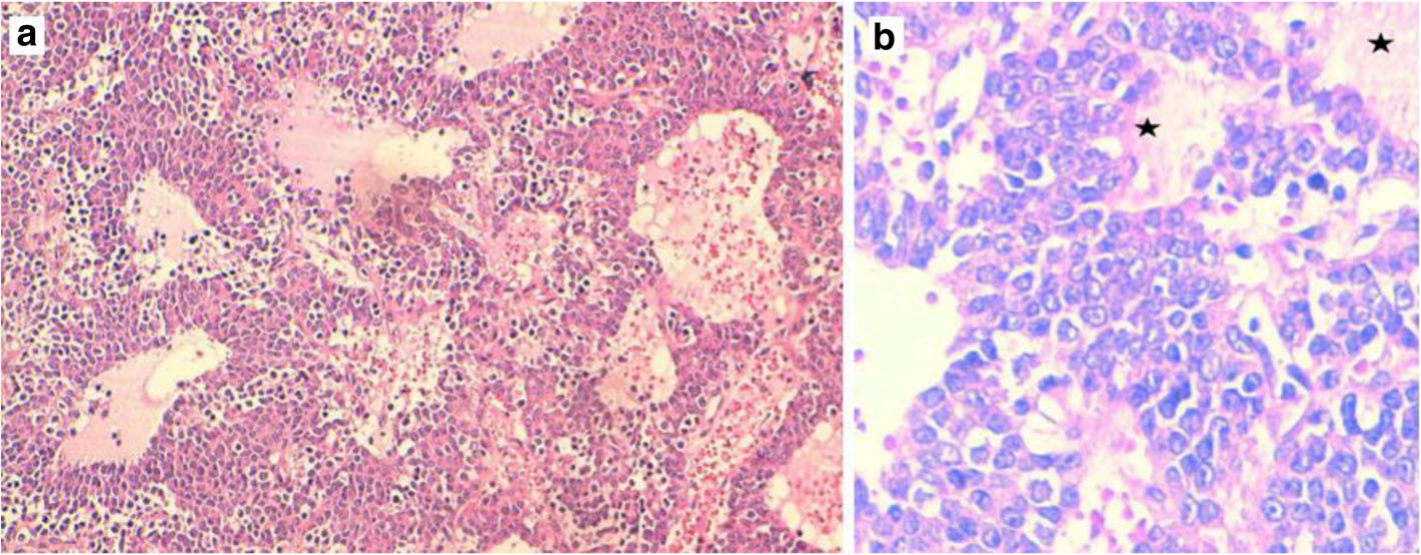
The histological view of the tumor shows a malignant proliferation disposed in nests and anastomosing trabecula with a rich vascular stroma. Tumor cells show palisading aspects around vascular structures; hematoxylin and eosin stain × 100 (**a**). At a higher magnification, the tumor cells are round to oval with irregular nuclei that have vesicular chromatin and conspicuous nucleoli. Foci of necrosis are associated (*black stars*); hematoxylin and eosin stain × 400 (**b**)

**Fig. 4:**
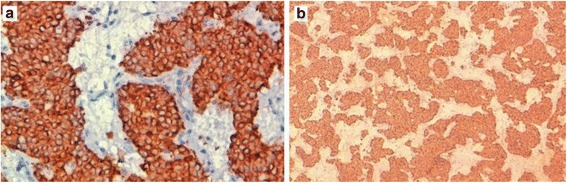
At immunohistochemistry, tumor cells stain strongly for cytokeratin AE1/AE3, ×400 (**a**) and chromogranin, ×100 (**b**)

**Fig. 5:**
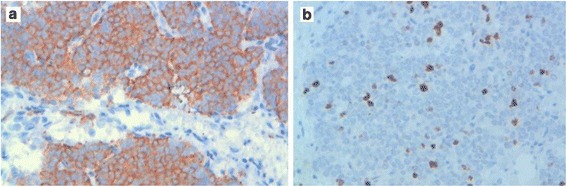
Tumor cells express CD117; ×400 (**a**). The Ki-67 proliferation index is estimated at 20%; ×400 (**b**)

A histological examination of the submitted biopsy specimen found a malignant proliferation disposed in nests and anastomosing trabecula within a fibrovascular stroma. The tumor cells were round to oval with irregular nuclei that had vesicular chromatin and conspicuous nucleoli. The tumor cells showed palisading aspects around vascular structures. Large foci of necrosis were also found (Fig. 3a, b). The mitoses count was 31 mitoses/2 mm^2^. At immunohistochemistry, tumor cells strongly expressed cytokeratin (CK) AE1/AE3, synaptophysin, chromogranin, and CD117. The Ki-67 proliferation index was 20% (Figs. 4 and 5). Tumor cells were negative for CK7, CK5/6, thyroid transcription factor-1 (TTF-1), CD20, CD3, CD5, terminal deoxynucleotidyl transferase (TdT), and CD99. The diagnosis of thymic LCNEC was made. At a multidisciplinary meeting (MDM), treatment of three cycles of carboplatin-paclitaxel chemotherapy was decided before an eventual surgery given the locally advanced stage of the tumor. At present, 3 months after the diagnosis, three cycles of chemotherapy has been prescribed and our patient is still alive in acceptable general condition.

## Discussion

NETs of the thymus are uncommon neoplasms accounting for less than 5% of all thymic neoplasms [1, 2]. The majority of these tumors are carcinoid tumors whereas high grade tumors are rare. Typically, thymic NETs occur in patients around 50 years of age. Thymic carcinoid tumors unlike their pulmonary counterparts affect mainly male patients. The high grade tumors (SCNEC and LCNEC) are less often encountered in men compared to carcinoid tumors [1, 6, 7]. Another different aspect is that thymic NETs are not associated with tobacco smoking whereas lung high grade tumors (SCNEC and LCNEC) are highly associated with tobacco [1]. Our current case of LCNEC had no tobacco smoking history and was a woman of 28 years showing that although it is a rare event, these tumors can affect young patients as reported in the literature [3, 7].

LCNECs as high grade tumors present mainly at an advanced clinical stage, with patients already having locally advanced disease or metastatic tumors. The related clinical signs are chest pain, cough, or dyspnea; locally advanced disease presents with signs of compression of neighboring structures such as lung, superior vena cava, heart, or nerve root leading to irradiating pain to arms or neck [6, 8, 9]. Exceptionally, patients with LCNEC present with paraneoplastic symptoms related to abnormal hormones production by the tumor such as adrenocorticotropic hormone (ACTH) with Cushing syndrome [1, 10]. The clinical presentation of our patient highlights the compression of mediastinal structures such us lung, heart, and nerve roots resulting in chest pain irradiating to her neck and her left arm. The imaging techniques showed mainly a tumor localized in the upper anterior mediastinum and its relationships with adjacent anatomical structures [11–13]. However, in the presence of a wide mediastinopulmonary tumor, like in our case, imaging techniques are challenged particularly by the issue of determining whether the tumor is a primary lung tumor, a thymic, or another mediastinal organ primary neoplasm. This issue is of the utmost importance especially for surgeons in order to perform adequate surgical management. However, in such a clinical presentation, often the only option is a thoracotomy or thoracoscopy approach in order to assess the macroscopic location and extension of the tumor, and the subsequent management can be performed with open biopsy for histological evaluation. On a CT scan, LCNEC of the lung generally has a peripheral location, rarely central, and bulky enlargement of the intrathoracic lymph node is uncommon [11, 13]. Thus, any large mass of the anterior mediastinum with lung involvement should indicate at first a diagnosis of primary thymic tumor invading the pulmonary parenchyma.

Thymic LCNEC is a high grade tumor composed of large cells with neuroendocrine features. Typically the tumor has organoid architecture and cells are arranged in nests and trabeculas with rosette-like formation. A delicate fibrovascular stroma is commonly associated. Areas of necrosis, usually extensive, are typically found within the tumor. LCNEC is characterized by large cells (more than the diameter of three resting lymphocytes) with granular to dark chromatin and conspicuous nucleoli. Frequent mitoses are characteristic, more than ten per 2 mm^2^ (an average of 45 per 2 mm^2^) [1–4]. At immunohistochemistry, LCNEC stains positive with neuroendocrine markers such as CD56, neuro-specific enolase, synaptophysin, and chromogranin. Keratins like CAM5.2 or AE1/AE3 are expressed by thymic LCNEC; cases that were positive for CD117 have been reported like our case [1, 14, 15]. Also, CD117 is useful to rule out the diagnosis of thymomas as these neoplasms are negative for this marker [15]. CD5 and TTF-1 are not expressed. As a high grade tumor, the Ki-67 proliferation index is high in LCNEC, around 40 to 80% [1].

The main differential diagnoses of thymic LCNECs are the other NETs of the thymus, thymomas, LCNEC of the lung invading the mediastinum, or basaloid squamous cell carcinoma. NETs of the thymus are classified by WHO into four main subtypes [1]. One subtype is carcinoid tumors, including the typical carcinoid that has less than two mitoses/2 mm^2^, without necrosis. Another type is the atypical carcinoid that has more mitotic count, two to ten mitoses/2 mm^2^, necrosis can be absent or present but not extensive like the other high grade NETs. SCNEC is a high grade tumor of the thymus that can pose a differential diagnosis with LCNEC. Both tumors have a high mitotic count, more than ten mitoses/2 mm^2^, and are characterized by the presence of extensive necrosis. The most discriminant features between the two tumors are the nuclear to cytoplasmic ratio and the presence or absence of nucleoli [1, 16]. LCNEC, by definition, is composed of large cells (more than the diameter of three resting lymphocytes) with prominent nucleoli. In SCNEC, tumor cells are smaller with inconspicuous nucleoli, if present. However, combined LCNEC exists with areas of SCNEC features [1]. Thymomas, of various types, especially in a small biopsy specimen can mimic some NET features (organoid architecture, rosette formation, prominent nucleoli). But careful clinical and minimal immunohistochemical analysis might establish the right diagnosis, as thymomas do not express commonly used neuroendocrine (synaptophysin and chromogranin) markers. Basaloid carcinoma of the thymus is another rare tumor that can mimic LCNEC morphology, with peripheral palisading and the presence of nucleoli in tumor cells. Basaloid carcinoma usually expresses p63 and p40, rarely CD5 and CK5/6, but is negative for neuroendocrine markers like synaptophysin and chromogranin [1]. Another more challenging issue is the differential diagnosis between primary thymic LCNEC extended to lung parenchyma and a primary pulmonary LCNEC invading the mediastinum [1, 2]. Correlations between clinical, radiological, and histopathological findings should be carefully done to determine the correct primary site of the tumor. Mostly, pulmonary LCNEC (like SCNEC) is highly associated with heavy tobacco smoking, whereas thymic LCNEC is not. With imaging techniques (CT scan especially), primary lung LCNEC is usually peripherally located, rarely central, and invades the mediastinal structures less often [11–13]. Thus, any anterior mediastinal LCNEC invading lung structures radiologically, suggests at first a locally advanced thymic primary tumor. With immunochemistry, TTF-1 and CK7 positive staining is highly suggestive of lung primary LCNEC. Recently, Weissferdt *et al*. in a study including 25 cases of pulmonary NETs and 25 cases of thymic NETs, concluded that PAX8+/TTF-1− immunophenotype appears to be more common in thymic neuroendocrine carcinomas, whereas the reverse (PAX8-/TTF-1+) is true for most pulmonary neuroendocrine carcinomas [2]. However, this study used only carcinoid tumors (low and intermediate grade tumors). Table 1 summarizes some differential features between lung LCNEC and thymic LCNEC. Our current case had no history of tobacco smoking, a CT scan and mediastinotomy confirmed the thymic location of the tumor, and immunohistochemistry ruled out the lung phenotype (TTF-1 and CK7 negative).

**Table 1:** Clinicopathological differential features of primary lung large cell neuroendocrine carcinoma and primary thymic large cell neuroendocrine carcinoma

	Lung LCNEC	Thymic LCNEC
Association with tobacco smoking	Yes	No
Imaging	Peripheral tumor, rarely invading mediastinal structures	Anterior mediastinal tumor
Immunohistochemistry:		
CK7	+	−
TTF-1	+	−
PAX8	−	+
CD117	+/−	+/−

*CK* cytokeratin, *LCNEC* large cell neuroendocrine carcinoma, *TTF-1* thyroid transcription factor-1, + usually positive, − usually negative

While pulmonary LCNEC displays inactivation mutations in the *RB* and *TP53* genes, with tobacco carcinogen-associated molecular signature (G-T transversion), thymic LCNEC does not carry this tobacco-related molecular signature [1].

As a high grade tumor, thymic LCNEC has a poor prognosis, with a reported 5-year overall survival ranging from 30 to 66% [1, 5, 9]. Complete surgical resection with or without chemotherapy seems to have a better outcome [9, 17]. The therapeutic modalities used in thymic NET are often those of lung NET; however, specific treatment for thymic tumors needs to be clearly defined [5, 17].

## Conclusions

Thymic LCNEC is a very rare tumor, with an aggressive course. Little is known about the clinical, epidemiological, and biological behavior of this uncommon neoplasm. More studies are needed to identify specific characteristics of thymic NET in order to design more suitable management.

## Abbreviations

CK: Cytokeratin
CT: Computed tomography
LCNEC: Large cell neuroendocrine carcinoma
NET: Neuroendocrine tumor
SCNEC: Small cell neuroendocrine carcinoma
TTF-1: Thyroid transcription factor-1
WHO: World Health Organization
